# The Potential of Infrared Thermography for Early Pregnancy Diagnosis in Nili-Ravi Buffaloes

**DOI:** 10.3390/ani14131966

**Published:** 2024-07-02

**Authors:** Umair Riaz, Musadiq Idris, Mehboob Ahmed, Farah Ali, Umer Farooq, Liguo Yang

**Affiliations:** 1 Hubei Hongshan Laboratory, Wuhan 430070, China; umair.riaz@iub.edu.pk (U.R.); mehboob.alyani@gmail.com (M.A.); 2 National Center for International Research on Animal Genetics, Breeding and Reproduction (NCIRAGBR), Ministry of Science and Technology, Huazhong Agricultural University, Wuhan 430070, China; 3 Department of Theriogenology, Faculty of Veterinary Science, The Islamia University of Bahawalpur, Bahawalpur 63100, Pakistan; drfarahiub@gmail.com; 4 Department of Physiology, Faculty of Veterinary and Animal Sciences, The Islamia University of Bahawalpur, Bahawalpur 63100, Pakistan; musadiq.idris@iub.edu.pk (M.I.); umer.farooq@iub.edu.pk (U.F.)

**Keywords:** buffalo, early pregnancy, infrared thermography, surface temperature

## Abstract

**Simple Summary:**

Early diagnosis of pregnancy in dairy buffaloes is of critical importance for harnessing their maximum production potential. Existing methods of early pregnancy diagnosis are highly technical, expensive, and laborious. Infrared thermography is emerging as a user-friendly, less laborious, and relatively simple technique that is capable of detecting variations in surface temperature associated with physiological changes in the animal’s body. The present study evaluated the potential of infrared thermography to detect thermal changes associated with early pregnancy in different anatomical regions (vulva, eyes, muzzle, flanks) in buffaloes. The results of the present study revealed that body-surface temperature as detected by infrared was significantly higher for the left flank (maximum, average, and minimum) and left eye and vulva (maximum and average gradients). The muzzle temperature at the maximum gradient was significantly higher for the non-pregnant as compared to pregnant buffaloes. The results indicated that various regions of the body tend to show differences in the surface temperature after pregnancy has been established and that infrared thermography could serve as a tool to measure such pregnancy-associated thermal changes.

**Abstract:**

This study was designed to explore the potential of infrared thermography (IRT) as an alternate approach for early pregnancy diagnosis in buffaloes. The surface temperature (ST) of different regions (eyes, muzzle, flanks, and vulva) was determined in 27 buffaloes using IRT from the day of artificial insemination (AI; Day 0), and measurement was repeated every fourth day until Day 24 post-AI. From all regions, the ST in each thermograph was recorded at three temperature values (maximum, average, minimum). Pregnancy status was confirmed through ultrasonography on Day 30, and animals were retrospectively grouped as pregnant or non-pregnant for analysis of thermographic data. In pregnant buffaloes, all three values of ST were significantly greater (*p* ≤ 0.05) for the left flank, while, in the left eye and vulva, only the maximum and average values were significantly greater. By contrast, the maximum ST of the muzzle was significantly lower (*p* ≤ 0.05) in pregnant buffaloes compared to non-pregnant buffaloes. However, the ST of the right eye and right flank did not show significant temperature variation at any value. These findings suggest that IRT has the potential to identify thermal changes associated with pregnancy in buffaloes at an early stage.

## 1. Introduction

Buffaloes hold significant importance as dairy animals, being the second-largest contributor to global milk production. To adequately harness the production potential of buffaloes, early identification of non-pregnant animals and their re-insemination are of paramount importance [1]. Delay in detecting the non-pregnant animals significantly escalates managerial and monetary inputs, jeopardizing the economic viability of rearing dairy buffaloes [2]. The significance of early pregnancy diagnosis is translated into improved reproductive efficiency, enhanced productivity, and competent farm economics, especially in the context of dairy-buffalo operations [1,3].

Currently, conventional methods employed for pregnancy diagnosis in buffaloes include rectal palpation [4], progesterone quantification in milk/blood [5,6], and ultrasonography [7]. These methods efficiently detect non-pregnant buffaloes around and after 25 days post-breeding, which is later than the next natural breeding opportunity, i.e., estrus (after an average of 21 days post-breeding). In addition to these traditional approaches, the detection of several biomarkers in maternal fluids such as pregnancy-associated glycoproteins [8], early pregnancy factors [9], urinary metabolites [10], and circulatory microRNAs [11,12] can be helpful in early pregnancy diagnosis in buffaloes. Unfortunately, these techniques demand a high level of technical expertise and are very expensive to use at a herd or farm level, limiting their adoption by farmers. Scientists are continuously working to find a practically convenient, relatively cheaper, non-invasive, and user-friendly method for early pregnancy estimation in buffalo.

Infrared thermography (IRT) is emerging as a potential tool for reproductive management, including estrus monitoring [13] and early pregnancy diagnosis [14] in farm animal species. IRT is handy for detecting and quantifying heat radiation emitting from the surface of an object. Various physiological (muscular activity, estrus, pregnancy, etc.) and pathological (stress, inflammations, pain, etc.) changes are associated with changes in the thermal status of the animal and warrant the use of this technique in veterinary practice [15,16,17,18,19]. These changes in body temperatures can be monitored through an infrared thermal camera. Major reproductive events like estrus and pregnancy are accomplished by the interplay of steroid hormones. These are responsible for changes in the blood flow to and from the genital organs. Progesterone has been reported to have a thermogenic property and, similarly, estrogen is known to induce hyperactivity and consequent temperature rise [13]. Considering these properties, IRT has been evaluated for estrus alerts [20], estimating the time of the luteinizing hormone (LH) surge and ovulation in animals [21,22]. Likewise, various studies have been conducted in different wild [23,24] and domesticated species to detect pregnancy and evaluate the progression of pregnancy [25,26,27,28]. Olğaç et al. [14] reported that the use of IRT is very promising for early pregnancy diagnosis in cattle. Buffaloes, due to their black skin color and less hairy coat, are assumed to possess better emissivity, as dark and smooth surfaces have a better ability to absorb and emit infrared radiation to maintain body temperature [13].

Therefore, it is hypothesized that IRT can be used to determine temperature variation arising possibly due to the difference in steroid-hormone profile and blood flow between pregnant and non-pregnant buffaloes at the time of insemination and during early pregnancy.

The present study is a pilot trial designed to evaluate the potential of IRT to detect surface-temperature variation at various anatomical regions (vulva, eyes, muzzle, flanks) between pregnant and non-pregnant buffaloes at the time of insemination and in early pregnancy.

## 2. Materials and Methods

### 2.1. Animals

The present study was conducted during the breeding season of the buffalo raised by small-scale livestock keepers (December and January 2021) in Bahawalpur, Pakistan. Twenty-seven 3.5-to-7-year-old lactating buffaloes (postpartum days, mean ± SE = 147.4 ± 17.5) of mixed parity that were clinically healthy with a body condition score of 3 ± 0.7 measured on a scale of 1 to 5 (1 = emaciated and 5 = obese) were selected as subjects in this study. Before enrollment in the trial, all buffaloes were examined by a trained veterinary professional and declared free of palpable abnormalities in their reproductive tract.

### 2.2. Experimental Design and Infrared Thermography

Following the occurrence of spontaneous estrus, all buffaloes were artificially inseminated by a trained technician with frozen semen sourced from a bull of known fertility. The day of artificial insemination (AI) was designated as ‘Day 0’ of the experiment. In all animals, before the AI, IRT was performed following the instructions and precautions explained earlier [13]. The infrared thermographic imaging continued at an interval of four days until ‘Day 24’ of the experiment in all animals. Thermal images were taken using an infrared thermal camera (Fluke PTi120 Pocket Thermal Imager with IR resolution 120 × 90, 1,800 pixels and temperature range −20 to 400 °C; Fluke Corporation, Everett, WA, USA) with emissivity set at 0.98. The anatomical regions of interest, specifically the vulva, eyes, muzzle, and flanks, were selected for taking thermal images, as they are prominent on the animal body surface and easily accessible for IRT imaging, having sufficient superficial blood circulation to dissipate metabolic heat to maintain homeostasis. It was ensured that the body parts of the animal subjected to thermographic imaging had clean surfaces without mud or dung present. Thermal images were taken for the above-mentioned anatomical regions in the morning at the same time every day, with the camera placed at an angle of 90° at a distance of approximately 1 m from the surface. All images were taken in the shade, and special attention was paid to avoiding physical activity by the animals before the thermal imaging. Images were transferred to the computer and were analyzed using software named ‘Fluke Connect (version 1.1.551.0)’. The maximum, average, and minimum temperatures were analyzed.

### 2.3. Pregnancy Diagnosis

Pregnancy status was confirmed 30 days after artificial insemination with the help of transrectal B-mode ultrasonography. A linear probe having a frequency of 7.5 MHz fitted with an ultrasound unit ‘Honda HS 200′ (Honda Electronics, Toyohashi, Japan) was used to scan the uterine horns and body transrectally. Buffaloes found to have an embryonic heartbeat (*n* = 11) were declared pregnant, while those with absence of an embryo (*n* = 16) were recorded as non-pregnant. Retrospectively, the thermographic data of the animals were grouped and analyzed separately based on pregnancy status.

### 2.4. Statistical Analyses

The data obtained from 27 Nili-Ravi buffaloes to explore the potential of IRT as an alternate approach for diagnosing early pregnancy were analyzed using the statistical software Minitab 19 (Minitab^®^ 19.1 Inc., Chicago, IL, USA). All data were analyzed for normality using the Anderson–Darling normality test. The data were analyzed using a generalized linear model (GLM) with the fixed factors Group, Day, and interaction between fixed factors (Group × Day), and post hoc comparisons were made with Tukey’s 95% test. The animals’ identification numbers were taken as a random factor. The statistical model can be described by the equation below:

Y_IRT(Body Surface)_ = µ + Group + Day + ID + (Group × Day) + e (1)

Here, Y_IRT(Body Surface)_ is the expected value of infrared body surface temperature; µ is the expected mean value for response variables, equal to zero, Group and Day are fixed factors, and e is the random error associated with experimental observations.

## 3. Results

The current study was planned to assess the potential of IRT as an alternate/quick approach for early pregnancy detection in buffaloes. The data representing variations in the body-surface temperature measured through IRT at different anatomical regions (left eye, right eye, muzzle, left flank, right flank, and vulva) of the pregnant and non-pregnant buffaloes are given in Table 1.

The maximum (*p* = 0.007) and average (*p* = 0.010) surface temperatures of the left eye were significantly higher in pregnant buffaloes compared to non-pregnant ones. However, the minimum surface temperature of the left eye did not vary significantly between groups (*p* = 0.107) (Table 1). No surface-temperature value of the right eye (maximum, average, or minimum) was statistically different between pregnant and non-pregnant buffaloes. Overall, the surface temperatures of the right and left eye did not differ significantly between the experimental groups on different experimental days (Group × Day; Table 1).

The maximum surface temperature of the muzzle was significantly higher (*p* = 0.027) in the non-pregnant buffaloes than in the pregnant animals; this result is opposite that observed for the left eye (Table 1). By contrast, the average and minimum surface temperatures of the muzzle exhibited no significant differences between the pregnant and non-pregnant buffaloes.

**Figure 1:**
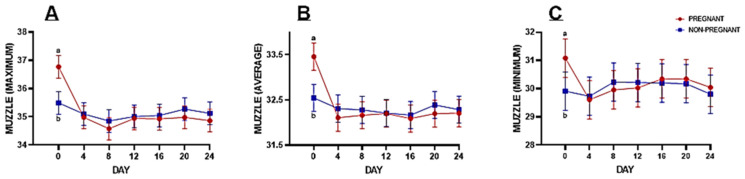
Fisher’s pairwise comparison of infrared surface temperature for the muzzle region at all three values, i.e., maximum (**A**), average (**B**), and minimum (**C**), in pregnant and non-pregnant buffaloes on different experimental days.

The interaction of the fixed variables (Group × Day) for the muzzle region revealed significantly higher surface-temperature maximum (*p* = 0.002), average (*p* = 0.009), and minimum (*p* = 0.049) values on the day of artificial insemination (Day 0) in pregnant animals compared to non-pregnant ones (Figure 1). However, during the remaining days of the experiment, i.e., Days 4, 8, 12, 16, 20, and 24, the difference in the infrared surface temperature of the muzzle between the two experimental groups remained non-significant.

**Figure 2:**
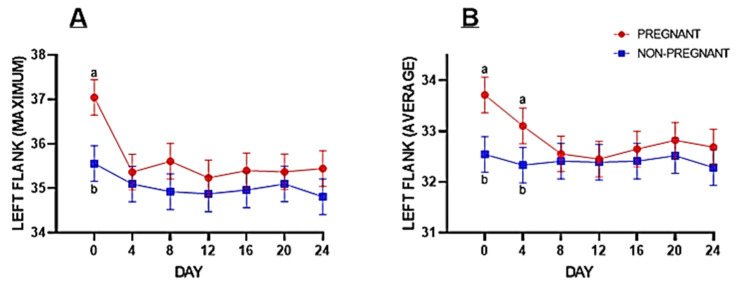
Fisher’s pairwise comparison of maximum (**A**) and average (**B**) infrared surface temperature of the left flank region in pregnant and non-pregnant buffaloes on different experimental days.

The infrared surface temperature of the left flank was significantly higher for pregnant buffaloes as compared to non-pregnant buffaloes at maximum (*p* = 0.013) and average (*p* = 0.010) values (Table 1). However, the minimum infrared surface temperature of the left flank was marginally higher (*p* = 0.055). The maximum surface temperature of the left flank was significantly higher on Day 0. A similar trend was observed in the average value on Day 0 and Day 4 in pregnant compared to non-pregnant buffaloes (Figure 2). The infrared surface temperature of the right flank was not statistically significant between experimental groups and on different experimental days between pregnant and non-pregnant animals (Table 1).

The maximum (*p* = 0.000) and average (*p* = 0.001) infrared surface temperatures at the vulva measured through IRT were significantly higher in pregnant buffaloes compared to the non-pregnant group. The minimum vulvar surface temperature did not show a significant difference between the two groups (pregnant and non-pregnant) of buffalo. However, the interaction between pregnancy group and the day of experiment was non-significant at all temperature values for the vulvar region.

## 4. Discussion

In buffaloes, detection of non-pregnant individuals at an early stage is necessary for maximum harnessing of the production potential of animals. This is a basic need for a dairy-based enterprise to make it economically viable [29]. Existing conventional methods give results after the next natural breeding opportunity (estrus) has passed, resulting in the wastage of crucial time and valuable resources [10]. Other identification techniques using early biomarkers are expensive and involve a lot of sampling; they are thus laborious and cause significant discomfort for operators and animals at the farm level. There is a need for a user-friendly, non-invasive, and less expensive technique that is helpful in identifying pregnancy status before the next natural breeding time.

Infrared thermography (IRT) is a non-invasive and user-friendly technique that is emerging as a potential tool for reproductive management. It is helpful in generating estrus alerts [13] and estimation of early pregnancy in dairy animals [14]. The main reproductive functions, including estrus and pregnancy, are controlled by the interplay of steroid hormones. These hormones are responsible for changes in the blood flow to and from the genital organs. Likewise, various studies have been conducted in different species to detect pregnancy and evaluate the progression of pregnancy [25,26,27]. The use of IRT is very promising for early pregnancy diagnosis in cattle [14]. The present study is a pilot trial designed to evaluate the potential of IRT to detect temperature differences between pregnant and non-pregnant buffaloes at the time of insemination and in early pregnancy.

In the current study, IRT was hypothesized to have greater potential to identify early pregnancy through thermal variation associated with pregnancy in buffaloes compared to cattle owing to their dark color and less hairy coat, which result in greater emissivity [13]. For this purpose, various anatomical regions of interest (vulva, eyes, muzzle, flanks) were selected, as they are prominent and easily accessible for thermal imaging on the animal body, having sufficient superficial blood circulation to dissipate metabolic heat to maintain homeostasis.

In the current study, the infrared thermal temperature of eyes (right and left) was observed, and the results indicated that the maximum and average surface temperatures of the left eye were significantly higher in pregnant buffaloes than in non-pregnant ones. On the contrary, no variation in mean surface temperature was observed for any value in the right eye. Shu et al. [30] stated that IRT is more reliable and repeatable in the left eye. Moreover, the surface temperature of the left eye has been reported to have a strong correlation with the core body temperature in cattle. In various reports, a higher core body temperature has been reported in pregnant animals than in non-pregnant animals. Gill et al. [31] reported that at the time point corresponding to embryo entry into the uterus, during the earlier stages of pregnancy, the core body temperature increased in pregnant cattle compared to non-pregnant cattle. Another study by Scanavez et al. 2017 [32] also reported an increase in vaginal temperature with the advancement of pregnancy in cattle. In the current study, the higher surface temperature of the left eye in pregnant buffaloes could be attributed to high core body temperature, as the left eye has been reported to be a better indicator of variation in the core body temperature compared to the right eye [30]. Physiological stresses like pregnancy, as well as the hypothalamic-pituitary axis, which controls the reproductive hormones, are governed by the right hemisphere of the brain. The left eye is connected to and represents the information processed in the right hemisphere [30]. Therefore, compared to the right eye, the left eye is assumed to better represent the thermographic changes associated with pregnancy.

In the present study, the maximum surface temperature of the muzzle was significantly lower in the pregnant group of buffaloes than in the non-pregnant group. This could be due to the maintenance of high progesterone levels in the buffaloes who conceived. In addition to its main function of pregnancy maintenance, progesterone is responsible for enhancing minute ventilation, predominantly due to an increase in tidal volume in pregnant animals [33]. Progesterone also causes sensitization of the primary respiratory system of the dam to carbon dioxide during pregnancy, leading to ventilatory changes [34,35]. A temperature rise due to increased progesterone and enhanced metabolic demand may cause an increase in respiration rate to dissipate internal heat [36,37]. Moreover, progesterone plays a crucial role in thermoregulation by modulating TRPV receptors. Specifically, progesterone has been shown to downregulate the expression and activity of TRPV4 channels, as demonstrated in human-airway and mammary-gland epithelial cells, as well as in vascular smooth-muscle cells [38]. This downregulation reduces calcium influx and channel currents, thereby affecting the heat-dissipation mechanisms. Consequently, the elevated progesterone levels during pregnancy could lead to higher localized temperatures in body parts such as the vulva because TRPV4 activity is suppressed, reducing the body’s ability to dissipate heat effectively. This mechanism supports our thermographic findings of increased body temperature in pregnant animals [38]. All these factors lead to moisturization of the muzzle surface due to evaporation and cause a drop in surface temperature at the muzzle. These explanatory factors were not considered practically in the present study and may be verified in the future in dedicated studies.

The results of the current study revealed that the surface temperature of the left flank was higher in pregnant compared to non-pregnant buffaloes, although the right flank region did not exhibit any difference in mean surface temperature between groups. This could be attributed to the rumen, which is a major organ under the left flank and is prone to temperature variations arising due to different physiological stages like estrus, the luteal phase, and pregnancy caused by the thermogenic property of progesterone [37]. In an earlier study, the temperature of the rumen in pregnant cows during the luteal phase (early pregnancy) was reported to be higher compared to that in non-pregnant cyclic cows as a result of enhanced metabolism, physiological changes to support fetal development, and increased feed intake [39,40].

The variation in the maximum and average surface temperatures of the vulva was greater in pregnant animals than in non-pregnant animals. This higher surface temperature could probably be due to the increase in blood supply to the reproductive tract that follows conception to deal with the increased needs of the dam to accommodate growing embryonic needs [41]. The blood flow to the genital organ increases during pregnancy owing to oxidative stress and enhanced metabolic demand [42]. The surface temperature of the tissue is representative of the underlying metabolic activity and changes in the blood flow that can be detected using IRT [43]. During estrus, an increase in vulvar temperature detected by IRT was reported to be associated with increased blood flow and was regarded as a very important factor in predicting estrus and/or ovulation [21,26]. There are reports that conclude, using Doppler ultrasonography, that blood flow to the genital tissues increases significantly during early pregnancy [44,45]. Therefore, the probable cause of increased vulvar temperature associated with the establishment of pregnancy in the current study might be associated with enhanced blood circulation to genital organs.

There was a significant increase in the surface temperature at the muzzle and left flanks on the day of insemination (Day 0) of animals subsequently identified as pregnant compared to those who failed to conceive (Figure 1). These findings are consistent with those reported earlier by Liles et al. [46], which stated that increased temperature at the time of insemination is positively correlated with pregnancy afterward in cattle. The chances of pregnancy increased 1.45 times with a 1 °C rise in body temperature. An elevated body temperature during estrus is important for resumption of meiotic activity in oocytes, which has functional significance during fertilization and pregnancy [46].

Previously various studies suggested the vulva and ocular region as good representatives of core body temperature during different physiological stages. However, the muzzle and flank were reported to be less well correlated with the core body temperature. Therefore, following the previous literature on preferred sites for IRT to monitor reproductive stages and the findings of the current pilot study, a systematic experiment is recommended.

The current study is a pilot study to evaluate the potential of infrared thermography as a tool to assess pregnancy-associated changes during early pregnancy in buffaloes. The study has limitations regarding the number of samples, and it is suggested that future studies should be conducted on a larger number of animals with more rigorous grouping. The current study made a comparison between pregnant and non-pregnant animals. However, all animals were inseminated, and it should be considered that possible cases of early/late embryonic death may have influenced the data gathered. Therefore, a more reliable control group should be created by including non-inseminated animals in the study.

## 5. Conclusions

This study revealed that the infrared body surface temperature was significantly higher for the left flank (maximum, average, and minimum) and left eye and vulva (maximum and average). The maximum muzzle temperature was significantly higher for the non-pregnant as compared to pregnant buffaloes. The results indicated that various regions of the body tend to show differences in the surface temperature after pregnancy has been established and that IRT could serve as a complementary tool to measure such pregnancy-associated thermal changes.

## Figures and Tables

**Table 1:** Infrared surface temperature of different anatomical regions of pregnant and non-pregnant buffaloes.

Parameter	Surface Temperature (°C)		SED	F-Value	*p*-Value		
	Pregnant	Non-Pregnant			Group	Day	Group × Day
Left eye maximum	35.8 ^a^	35.2 ^b^	0.61	7.40	0.007	0.686	0.190
Left eye average	33.2 ^a^	32.7 ^b^	0.50	6.74	0.010	0.613	0.550
Left eye minimum	30.5	30.1	0.59	2.63	0.107	0.504	0.954
Right eye maximum	35.8	35.8	0.66	0.01	0.920	0.730	0.539
Right eye average	33.1	33.1	0.51	0.06	0.806	0.669	0.606
Right eye minimum	30.5	30.4	0.57	0.44	0.509	0.005	0.667
Muzzle maximum	34.6 ^b^	35.2 ^a^	0.64	4.94	0.027	0.060	0.002
Muzzle average	32.2	32.5	0.59	1.88	0.172	0.002	0.009
Muzzle minimum	29.7	29.8	0.68	0.09	0.764	0.000	0.049
Left flank maximum	35.6 ^a^	34.9 ^b^	0.65	6.24	0.013	0.000	0.009
Left flank average	32.7 ^a^	32.2 ^b^	0.56	6.74	0.010	0.000	0.011
Left flank minimum	29.9 ^a^	29.4 ^b^	0.67	3.72	0.055	0.003	0.088
Right flank maximum	35.6	35.4	0.65	0.56	0.457	0.035	0.767
Right flank average	32.8	32.4	0.69	1.81	0.180	0.027	0.948
Right flank minimum	30.1	29.56	0.87	2.52	0.115	0.072	0.954
Vulva maximum	36.5 ^a^	35.3 ^b^	0.64	23.82	0.000	0.001	0.994
Vulva average	33.9 ^a^	33.1 ^b^	0.54	11.64	0.001	0.000	0.960
Vulva minimum	31.3	31.0	0.59	1.43	0.233	0.000	0.388

SED: standard error of the difference between two means; Means with different superscripts differ significantly (*p* ≤ 0.05) by Fisher’s pairwise comparisons.

## Data Availability

Data are contained within the article.

## References

[1] Balhara A.K., Gupta M., Singh S., Mohanty A.K., Singh I. (2013). Early pregnancy diagnosis in bovines: Current status and future directions. Sci. World J..

[2] Yadav R., Tripathi H., Kumar P., Ramesh N. (2019). Assessing the economic losses to buffalo owners due to late diagnosis of pregnancy in their milch buffaloes. AJAEES.

[3] Barile V.L., Menchetti L., Casano A.B., Brecchia G., Melo de Sousa N., Zelli R., Canali C., Beckers J.F., Barbato O. (2021). Approaches to identify pregnancy failure in buffalo cows. Animals.

[4] Karen A.M., Darwish S., Ramoun A., Tawfeek K., Van Hanh N., de Sousa N.M., Sulon J., Szenci O., Beckers J.-F. (2011). Accuracy of transrectal palpation for early pregnancy diagnosis in Egyptian buffaloes. Trop. Anim. Health Prod..

[5] Perera B., Pathiraja N., Abeywardena S., Motha M., Abeygunawardena H. (1980). Early pregnancy diagnosis in buffaloes from plasma progesterone concentration. Vet. Rec..

[6] Singh A., Puthiyandy R. (1980). Estimation of progesterone in buffalo milk and its application to pregnancy diagnosis. Reproduction.

[7] Karen A., Darwish S., Ramoun A., Tawfeek K., Van Hanh N., De Sousa N., Sulon J., Szenci O., Beckers J.-F. (2007). Accuracy of ultrasonography and pregnancy-associated glycoprotein test for pregnancy diagnosis in buffaloes. Theriogenology.

[8] Barbato O., Barile V.L. (2023). Pregnancy-associated glycoproteins in buffalo: Origins, functions and clinical application for pregnancy follow-up. Rev. Cient. Fac. Vet..

[9] Mavedati O., Rastegarnia A., Habibian R., Bari Y.N., Bandarian E. (2013). Early pregnancy diagnosis in water buffalo by early pregnancy factor measurement using rosette inhibition test. Glob. Vet..

[10] Sarangi A., Ghosh M., Sangwan S., Kumar R., Balhara S., Phulia S., Sharma R., Sahu S., Kumar S., Mohanty A. (2022). Exploration of urinary metabolite dynamicity for early detection of pregnancy in water buffaloes. Sci. Rep..

[11] Guelfi G., Stefanetti V., De Luca S., Giontella A., Barile V.L., Barbato O. (2017). Serum microRNAs in buffalo cows: Potential biomarkers of pregnancy. Res. Vet. Sci..

[12] Sarwalia P., Raza M., Soni A., Dubey P., Chandel R., Kumar R., Kumaresan A., Onteru S.K., Pal A., Singh K. (2021). Establishment of Repertoire of Placentome-Associated MicroRNAs and Their Appearance in Blood Plasma Could Identify Early Establishment of Pregnancy in Buffalo (*Bubalus bubalis*). Front. Cell Dev. Biol..

[13] Riaz U., Idris M., Ahmed M., Ali F., Yang L. (2023). Infrared Thermography as a Potential Non-Invasive Tool for Estrus Detection in Cattle and Buffaloes. Animals.

[14] Olğaç K.T., Yazlık M.O., Kaya U., Özkan H., Tırpan M.B. (2023). The thermographic monitoring in early pregnancy detection in Holstein cows and heifers. Anim. Reprod. Sci..

[15] Rekant S.I., Lyons M.A., Pacheco J.M., Arzt J., Rodriguez L.L. (2016). Veterinary applications of infrared thermography. Am. J. Vet. Res..

[16] Zhang C., Xiao D., Yang Q., Wen Z., Lv L. (2020). Application of Infrared Thermography in Livestock Monitoring. Trans. ASABE.

[17] Ghezzi M.D., Ceriani M.C., Domínguez-Oliva A., Lendez P.A., Olmos-Hernández A., Casas-Alvarado A., Hernández-Avalos I. (2024). Use of Infrared Thermography and Heart Rate Variability to Evaluate Autonomic Activity in Domestic Animals. Animals.

[18] Whittaker A.L., Muns R., Wang D., Martínez-Burnes J., Hernández-Ávalos I., Casas-Alvarado A., Domínguez-Oliva A., Mota-Rojas D. (2023). Assessment of pain and inflammation in domestic animals using infrared thermography: A narrative review. Animals.

[19] Casas-Alvarado A., Mota-Rojas D., Hernández-Ávalos I., Mora-Medina P., Olmos-Hernández A., Verduzco-Mendoza A., Reyes-Sotelo B., Martínez-Burnes J. (2020). Advances in infrared thermography: Surgical aspects, vascular changes, and pain monitoring in veterinary medicine. J. Therm. Biol..

[20] Marquez H.P., Ambrose D., Schaefer A., Cook N., Bench C. (2021). Evaluation of infrared thermography combined with behavioral biometrics for estrus detection in naturally cycling dairy cows. Animal.

[21] Talukder S., Kerrisk K., Ingenhoff L., Thomson P., Garcia S., Celi P. (2014). Infrared technology for estrus detection and as a predictor of time of ovulation in dairy cows in a pasture-based system. Theriogenology.

[22] Marquez H.P., Ambrose D., Schaefer A., Cook N., Bench C. (2019). Infrared thermography and behavioral biometrics associated with estrus indicators and ovulation in estrus-synchronized dairy cows housed in tiestalls. J. Dairy Sci..

[23] Hilsberg-Merz S. (2008). Infrared thermography in zoo and wild animals. Zoo Wild Anim. Med. Curr. Ther..

[24] Krueger F., Knauf-Witzens T., Getto S. (2019). New approach in thermal pregnancy diagnosis: Teat’s heating in babirusa (*Babyrousa babyrussa*). Theriogenology.

[25] Domino M., Borowska M., Kozłowska N., Zdrojkowski Ł., Jasiński T., Smyth G., Maśko M. (2021). Advances in thermal image analysis for the detection of pregnancy in horses using infrared thermography. Sensors.

[26] Radigonda V.L., Pereira G.R., da Cruz Favaro P., Barca Júnior F.A., Borges M.H.F., Galdioli V.H.G., Júnior C.K. (2017). Infrared thermography relationship between the temperature of the vulvar skin, ovarian activity, and pregnancy rates in Braford cows. Trop. Anim. Health Prod..

[27] Bowers S., Gandy S., Anderson B., Ryan P., Willard S. (2009). Assessment of pregnancy in the late-gestation mare using digital infrared thermography. Theriogenology.

[28] Kul H., Erdoğan G. (2022). Evaluation of infrared thermography findings in pseudopregnant rabbit. Anim. Health Prod. Hyg..

[29] Cardoso Consentini C.E., Wiltbank M.C., Sartori R. (2021). Factors that optimize reproductive efficiency in dairy herds with an emphasis on timed artificial insemination programs. Animals.

[30] Shu H., Li Y., Fang T., Xing M., Sun F., Chen X., Bindelle J., Wang W., Guo L. (2022). Evaluation of the best region for measuring eye temperature in dairy cows exposed to heat stress. Front. Vet. Sci..

[31] Gil Z., Kural J., Szarek J., Wierzchoś E. (2001). Increase in milk and body temperature of cows as a sign of embryo entry into the uterus. Theriogenology.

[32] Scanavez A., Fragomeni B., Rocha L., Voelz B., Hulbert L., Mendonça L. (2017). Association between 4-day vaginal temperature assessment during the dry period and performance in the subsequent lactation of dairy cows during the warm season. J. Anim. Sci..

[33] Keith I., Bisward G., Manohar M., Klein J., Bullard V. (1982). Respiratory effects of pregnancy and progesterone in Jersey cows. Respir. Physiol..

[34] LoMauro A., Aliverti A. (2015). Respiratory physiology of pregnancy: Physiology masterclass. Breathe.

[35] Bayliss D.A., Millhorn D.E., Gallman E.A., Cidlowski J.A. (1987). Progesterone stimulates respiration through a central nervous system steroid receptor-mediated mechanism in cat. Proc. Natl. Acad. Sci. USA.

[36] Noya A., Casasús I., Ferrer J., Sanz A. (2019). Long-term effects of maternal subnutrition in early pregnancy on cow-calf performance, immunological and physiological profiles during the next lactation. Animals.

[37] Suthar V., Burfeind O., Bonk S., Dhami A., Heuwieser W. (2012). Endogenous and exogenous progesterone influence body temperature in dairy cows. J. Dairy Sci..

[38] Jung C., Fandos C., Lorenzo I.M., Plata C., Fernandes J., Gené G.G., Vázquez E., Valverde M.A. (2009). The progesterone receptor regulates the expression of TRPV4 channel. Pflügers Arch.-Eur. J. Physiol..

[39] Kendall P., Webster J. (2009). Season and physiological status affects the circadian body temperature rhythm of dairy cows. Livest. Sci..

[40] Kim D.H., Ha J.J., Yi J.K., Kim B.K., Kwon W.-S., Ye B.-H., Kim S.H., Lee Y. (2021). Differences in ruminal temperature between pregnant and non-pregnant Korean cattle. J. Anim. Reprod. Biotechnol..

[41] Abouelela Y.S., Yasin N.A., Khattab M.A., El-Shahat K., Abdelnaby E.A. (2021). Ovarian, uterine and luteal hemodynamic variations between pregnant and non-pregnant pluriparous Egyptian buffalos with special reference to their anatomical and histological features. Theriogenology.

[42] Elmetwally M.A., Elshopakey G.E., Eldomany W., Eldesouky A., Samy A., Lenis Y.Y., Chen D.b. (2021). Uterine, vaginal and placental blood flows increase with dynamic changes in serum metabolic parameters and oxidative stress across gestation in buffaloes. Reprod. Domest. Anim..

[43] Nääs I.A., Garcia R.G., Caldara F.R. (2020). Infrared thermal image for assessing animal health and welfare. J. Anim. Behav. Biometeorol..

[44] Hassan M., Arshad U., Bilal M., Sattar A., Avais M., Bollwein H., Ahmad N. (2019). Luteal blood flow measured by Doppler ultrasonography during the first three weeks after artificial insemination in pregnant and non-pregnant Bos indicus dairy cows. J. Reprod. Dev..

[45] Samir H., Kandiel M.M. (2019). Accuracy of subjective evaluation of luteal blood flow by color Doppler ultrasonography for early diagnosis of pregnancy in Egyptian buffalo. Anim. Reprod. Sci..

[46] Liles H.L., Schneider L.G., Pohler K.G., Oliveira Filho R.V., Neal Schrick F., Payton R.R., Rhinehart J.D., Thompson K.W., McLean K., Edwards J.L. (2022). Positive relationship of rectal temperature at fixed timed artificial insemination on pregnancy outcomes in beef cattle. J. Anim. Sci..

